# Post-Covid-19 Syndrome: Improvements in Health-Related Quality of Life Following Psychology-Led Interdisciplinary Virtual Rehabilitation

**DOI:** 10.1177/21501319211067674

**Published:** 2021-12-23

**Authors:** Sari Harenwall, Suzanne Heywood-Everett, Rebecca Henderson, Sherri Godsell, Sarah Jordan, Angela Moore, Ursula Philpot, Kirsty Shepherd, Joanne Smith, Amy Rachel Bland

**Affiliations:** 1 Bradford District Care NHS Foundation Trust, Bradford, UK; 2 Manchester Metropolitan University, Manchester, UK; 3 Leeds Beckett University, Leeds, UK; 4 University of Bradford, Bradford, UK

**Keywords:** post-covid-19 syndrome, long-COVID, EuroQoL, interdisciplinary, rehabilitation

## Abstract

Coronavirus disease 2019 (COVID-19) is increasingly recognized as having significant long-term impact on physical and mental health. The Primary Care Wellbeing Service (PCWBS) in Bradford District Care NHS Foundation Trust (BDCFT) is a psychology-led specialist interdisciplinary team of health professionals specializing in persistent physical symptoms (PPS) and Chronic Fatigue Syndrome (CFS)/Myalgic Encephalomyelitis (ME) with an emphasis on holistic integrated care. The PCWBS quickly recognized the risk of the long-term effects of COVID-19, particularly for social, health and care staff, and developed a 7-week virtual rehabilitation course which was piloted in October 2020. The “*Recovering from COVID*” course takes a whole system, biopsychosocial approach to understanding COVID-19 and post-viral fatigue (PVF) and is delivered by an interdisciplinary team consisting of a clinical psychologist, physiotherapist, occupational therapist, dietitian, speech and language therapist, assistant psychologist, and a personal support navigator with support from a team administrator. The course focuses on understanding PVF, sleep optimization, nutrition, swallowing, activity management, energy conservation, stress management, breathing optimization, managing setbacks, and signposting to appropriate resources and services. Since the pilot, PCWBS has delivered 7 courses to support over 200 people suffering from post-COVID-19 syndrome. One hundred and forty-nine individuals that enrolled on the “*Recovering from COVID*” course completed the EQ-5D-5L to assess Health-related quality of life (HRQoL) across 5 dimensions, including problems with mobility, self-care, usual activities, pain/discomfort, and anxiety/depression. Subsequently, 76 individuals completed these measures at the end of the rehabilitation course showing that patient ratings were significantly improved. In response to the NIHR recommendation for rapid evaluation of different service models for supporting people with post-COVID-19 syndrome, this data offers hope that rehabilitation is effective in reversing some of the problems faced by people living with the long-term effects of COVID-19.

## Background

There is increasing evidence to suggest that coronavirus disease 2019 (COVID-19) caused by severe acute respiratory syndrome coronavirus 2 (SARS-CoV-2), can have significant long-term effects on health. Although most individuals recover from COVID-19 within weeks, it is now well established that some will present with persistent, long-term symptoms.^1^ Persistent symptoms may develop during or following the acute infection, with those lasting for more than 12 weeks commonly referred to as “long COVID” or post-COVID-19 syndrome.^2^ Post-COVID-19 Syndrome can occur regardless of whether the individual experienced severe, mild, and even no symptoms during acute COVID-19 infection^3^ and research suggests that patients with uncomplicated COVID-19 have long-term persistent symptoms and functional impairment similar to patients with severe COVID-19.^4^

The National Institute for Health and Care Excellence (NICE) describes post COVID-19 syndrome as a set of persistent physical, cognitive, and/or psychological symptoms that continue for more than 12-weeks following the acute illness and are not explained by an alternative diagnosis. A recent study of 508 707 participants found that 37.7% of participants reported at least one persistent symptom for 12 weeks following acute infection, with 14.8% reporting at least three symptoms.^5^ The incidence of post-COVID-19 syndrome is estimated at 10% to 35%, and for hospitalized patients, it has been reported to reach 85%.^6^ The Office for National Statistics (ONS) estimates 970 000 people in the UK are currently living with post-COVID-19 syndrome, with 66% of those reporting impacts to usual daily activities.^7^

The exact symptomatology of post COVID-19 syndrome is unknown, with one systematic review identifying 84 symptoms.^1^ Post-COVID-19 syndrome usually presents with clusters of symptoms, often overlapping, which can fluctuate and change over time and can affect any system in the body.^2^ Frequently reported symptoms include fatigue, breathlessness, cognitive impairment, insomnia, anxiety, and depression.^8^ These persistent symptoms have a significant impact on individuals, that exhibit reduced quality of life, alongside reduced capacity to work, and perform usual daily activities.^9^ Indeed, Davis et al^10^ found that only 27.3% of participants with current post-COVID-19 syndrome symptoms had resumed their usual working hours, with 45.6% working reduced hours and 23.3% not working as a direct result of their symptoms. Together, these findings suggest post-COVID-19 syndrome has a range of individual, social, and economic impacts, therefore underscoring the need to develop effective treatment options.

The underlying pathophysiology of post-COVID-19 syndrome is still relatively unknown, but is likely to involve multiple systems, including the central and autonomic nervous systems, immune system, respiratory system, musculoskeletal, and cardiovascular systems.^11,12^ This is unsurprising given that in the acute phase, SARS-CoV-2 has been found to impact multiple organ systems.^13^ With the heterogeneity of symptoms, attempts have been made to group symptoms into clusters: neurological/neuromuscular, musculoskeletal, gastrointestinal, neurocognitive, psychological/social, upper respiratory, cardiopulmonary, and systemic.^14^ Together, these findings support the assertion that post-COVID-19 syndrome is a multisystem condition, requiring a multisystem approach to its management.^15^

## Management of Post-COVID-19 Syndrome

Several recommendations have been made for the management of post-COVID-19 syndrome in primary care. The current NICE guidelines^2^ advocate a multidisciplinary approach to rehabilitation that supports individuals to self-manage their symptoms and resume day-to-day activities. However, there is currently little empirical evidence to guide the development of such rehabilitation services, particularly for patients presenting with non- urgent but debilitating symptoms, such as fatigue, post-exertional malaise, breathlessness, and cognitive impairment.

The ME association published a document in May 2020 warning of a potential surge in Post Viral Fatigue (PVF), a commonly reported symptom following an acute virus which lasts for several months^16^ and CFS/ME.^17^ Whilst exact etiology remains unknown, and future research is needed, it is now recognized that overlapping symptomology between post-COVID- 19 syndrome and ME/CFS provides a promising avenue for the development of post-COVID-19 rehabilitation. Wong and Weitzer^18^ investigated the potential connection between the 2 conditions, comparing post-COVID-19 symptoms reported across 21 studies with recognized ME/CFS symptomology. This analysis revealed a significant overlap in 25 out of 29 symptoms with the three major ME/CFS symptoms (fatigue, reduced daily activity, and post-exertional malaise) widely reported in the post-COVID-19 syndrome literature. Additionally, Twomey et al^9^ found the most prominent symptoms of post-COVID-19 syndrome were chronic fatigue and post-exertional malaise, with 71.4% of participants reporting chronic fatigue and 58.7% meeting the diagnostic criteria for ME/CFS. In response to the growing demand for effective rehabilitation for the long-term effects of COVID-19, the ME Association published guidelines to support recovery from post-viral fatigue syndrome following COVID-19 infection.^17^ The guidelines recommend individuals manage their activity to reduce post-exertional malaise, balancing activity with rest, in addition to managing their mental wellbeing, nutrition, and sleep.

A recent NIHR review^19^ predicts that existing NHS services will be unable to meet the needs of people with post-COVID-19 syndrome without developing new delivery models that allow rapid access, and furthermore recommends rapid evaluation of different service models for supporting people with post-COVID-19 syndrome. This manuscript aims to address this paucity of research by evaluating an interdisciplinary rehabilitation pathway delivered by the Primary Care Wellbeing Service (PCWBS) in Bradford District Care NHS Foundation Trust (BDCFT).

## Primary Care Wellbeing Service, Bradford District Care NHS Foundation Trust

The PCWBS is a psychology-led specialist interdisciplinary team of health professionals specializing in the holistic and integrated care of mental and physical health symptoms, with a particular focus on persistent physical symptoms (PPS) and CFS/ME. The PCWBS shared concerns outlined by the ME association^17^ of a potential surge in PVF and CFS/ME and recognized that offering early intervention to frontline workers was vital. Previously, similar viral outbreaks such as SARS in Hong Kong in 2003 showed subsequent clusters of ME/CFS, that were present even at a 4-year follow-up.^20^ Therefore, the PCWBS’s rapid response was to provide care, knowing that even if people with COVID-19 appear to recover, they could face lifelong risks and disability.

## “*Recovering From COVID*” 7-Week Rehabilitation Course

**Figure 1.**
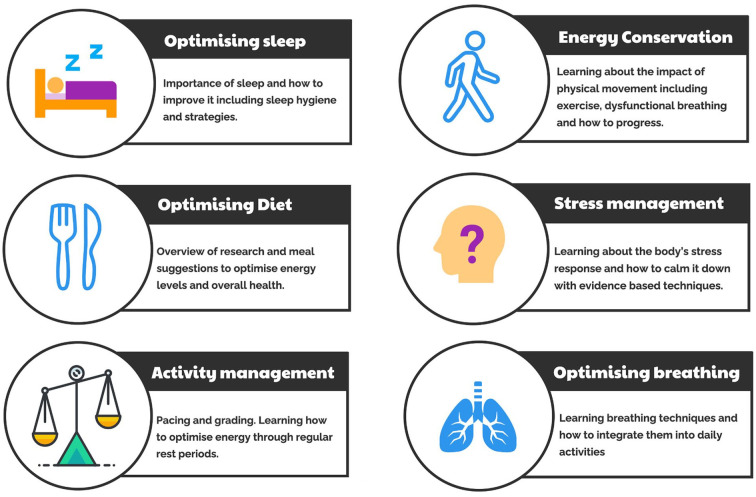
The PCWBS “*Recovering from COVID*” 7-week rehabilitation course focusing on optimizing sleep, diet, activity management, energy conservation, stress management, and optimizing breathing.

The “*Recovering from COVID*” course is a 7-week course offering support to all social, health, and care staff working in Bradford, Airedale, and Craven who are experiencing long-term symptoms of COVID-19. Using expertise in CFS/ME rehabilitation, the specialized, interdisciplinary team developed a rehabilitation program tailored to post-COVID-19 syndrome (Figure 1). PCWBS offered the first 7-week rehabilitation course in October 2020 and have subsequently ran a further 7 courses, delivered virtually using Microsoft Teams, supporting over 200 people suffering from post-COVID-19 syndrome. The course takes a whole system, biopsychosocial approach to understanding COVID-19 and post-viral fatigue and is led by an interdisciplinary team consisting of a clinical psychologist, physiotherapist, occupational therapist, dietitian, speech and language therapist, and a personal support navigator. Participants received a workbook to consolidate learning and develop skills to enhance and encourage the process of rehabilitation. As an integral part of the 7-week course, various exercises are demonstrated and practiced each week, with participants encouraged to incorporate them into their daily routines using recognized behavior change techniques (BCTs), such as self-monitoring, action planning, and problem solving.^21^ The exercises include relaxation, breathing and mindfulness exercises, as part of promoting activation of the parasympathetic nervous system.

Each session lasted 1 h, and started at 1 pm based on the assumption that those who were back at work would be supported to attend whilst not competing with core childcare hours. Sessions were not recorded due to confidentiality, but all resources were shared with those that missed a session. Participants were encouraged to contact the team for debrief/wellbeing support if anyone became distressed during the course and were subsequently sign-posted appropriately.

### Week 1: Understanding COVID-19 and Post Viral Fatigue

The first week of the course, facilitated by a clinical psychologist and physiotherapist or occupational therapist, provides participants with an overview of the existing research concerning post COVID-19 syndrome, describing the myriad of symptoms that may be experienced and the links to PVF and ME/CFS. The facilitator introduces key theories relevant to the development of the rehabilitation pathways, such as the proposed role of mitochondria in ME/CFS^22^ and polyvagal theory.^23^ Participants were educated in the concept of a “whole systems” approach to recovery, to promote a superordinate framework from the start, emphasizing the relationship between sleep, mood, breathing pattern, diet, and activity. Further education was provided to link this approach to an understanding of the brain and body, and their natural response to physical and psychological stress.

Participants are encouraged to share their experiences of post-COVID-19 syndrome, enabling facilitators to provide tailored advice to support individual recovery. Brief education is offered on dysfunctional breathing patterns and a breathing exercise is practiced. Participants are encouraged, within their own limits, to continue this exercise frequently during their recovery. The accompanying booklet contains further information about ME/CFS, building participants’ understanding of the factors that may facilitate or impede their recovery. Homework tasks support participants to explore their symptoms in more detail and prioritize those causing the most difficulty. Participants are also encouraged to complete a sleep diary prior to the second session.

### Week 2: Importance of Sleep and How to Improve It

The second week, facilitated by a clinical psychologist and physiotherapist or occupational therapist, focuses on the importance of sleep and evidence-based strategies for management. Sleep disturbance is widely reported in both post COVID-19 syndrome and ME/CFS.^24^ These symptoms include insomnia and hypersomnia, with many participants reporting fatigue upon waking.^25,26^ Therefore the inclusion of strategies to optimize sleep may enhance the efficacy of post-COVID rehabilitation services.^25^ The session begins with a group discussion, in which participants are encouraged to discuss sleep problems, changes to their sleep patterns, and quality of sleep. The course facilitators provide an overview of sleep and the circadian rhythm, how it may be disrupted by COVID-19, and the factors that may maintain or exacerbate sleep disturbance. Strategies for optimizing sleep and managing nightmares are subsequently described, with a list of resources provided, including an online video to advise on the self-management of nightmares.^27^ The associated workbook elaborates upon the information provided in the session, supporting participants to establish their baseline and make small changes based upon the sleep diary they completed. Participants are also encouraged to decide upon one change they will make to their sleep routine, create an action plan for this change, and consider any barriers that may arise during its implementation. To prepare for the next session, participants are encouraged to maintain a food diary.

### Week 3: Diet and Voice

The third session is facilitated by a dietitian and speech and language therapist, that cover aspects of COVID-19 rehabilitation related to diet and communication. The first part of the session focuses on diet, describing several key areas of nutrition relevant to post COVID- 19 syndrome symptoms, such as weight loss, reduced appetite, and fatigue. The facilitator describes research that suggests high blood glucose levels may be associated with severe COVID-19^28^ and the role nutrition may play in recovery from post COVID-19 syndrome. Strategies are provided for optimizing nutrition to meet post-COVID-19 health needs, including specific micronutrients, supplementation recommendations, and blood glucose management. Support is also provided to those experiencing changes to taste and smell, with olfactory training advised.^29^

The second part of the session focuses on areas relevant to speech and language therapy, including communication and swallowing. Practical advice is provided to assist participants to rehabilitate inflamed vocal cords and resolve problems impeding swallowing. Workbooks are provided to elaborate upon the issues addressed in the session, with a range of activities intended to guide participants to implement recommended changes. Participants are encouraged to complete an activity log in preparation for the fourth session.

### Week 4: Activity Management

The fourth session addresses activity management and is facilitated by an occupational therapist and clinical psychologist. As is the case with patients with ME/CFS, individuals with post COVID-19 syndrome may also benefit from developing an energy management plan with an interdisciplinary team including understanding the patients’ activity threshold and managing daily energy expenditure in order to maintain a healthy active lifestyle while reducing symptom flare-up.^19^ This part of the course explains the “boom-and-bust” pattern of activity characteristic of ME/CFS^30^ and guides participants to establish their baseline patterns of fatigue and categorize activities according to their energy requirements. Indeed, some patients report worsening of post-COVID-19 syndrome following periods of increased activity.^31^ Consequently, the course aims to support individuals to balance periods of activity with rest to conserve energy and avoid symptom exacerbation.^32^ The facilitator introduces participants to the key principles of activity management, such as pacing, which has been found to promote a return to daily activities in ME/CFS, while reducing relapses and post-exertional malaise.^33^ The workbook provides further information to guide these activities, supporting the development of an activity management action plan and encourage the use of relaxation strategies. Participants are encouraged to keep an activity diary to self-monitor their progress and identify triggers.

### Week 5: Movement and Energy Conservation

The fifth session is facilitated by a physiotherapist and further considers the role of physical activity in recovery from post-COVID-19 syndrome, explaining how prolonged periods of inactivity may lead to physical and cardiovascular deconditioning.^34^ Participants are encouraged to closely monitor their activity and symptoms, listening to their body in order to gently build up their tolerance for physical activity while adhering to the principles of activity management (eg, pacing and energy conservation) covered in week 4. Participants are encouraged to reduce activity as required and to only proceed with increases in activity following a period of stability. An analogy, presenting recovery as a rollercoaster ride, is used to demonstrate that ups and downs are to be expected. The session also addresses prevalent post-COVID-19 syndrome symptoms that may impede physical activity, such as dysfunctional breathing patterns, breathlessness and persistent coughing, introducing strategies to help participants self-manage these symptoms and resume physical activity. The workbook provides further practical guidance for managing physical activity, supporting participants to recognize signs that activity has been increased too quickly and to develop an action plan to deal with setbacks.

### Week 6: Stress Management

The sixth session is facilitated by a clinical psychologist and addresses the emotional impact of COVID-19, the role of the stress response, and strategies for managing stress. Stress intolerance has been reported in post-COVID-19 syndrome,^35^ which aligns with previous research that suggests ME/CFS symptoms may be exacerbated by stress.^36^ Additionally, research from the field of psychoneuroimmunology suggests that symptoms such as fatigue and cognitive dysfunction may be attenuated by stress management,^37^ situating stress management as an important target in post-COVID-19 syndrome rehabilitation.

Participants are invited to share their psychological and emotional experiences relating to COVID-19, before learning about the role of the autonomic nervous system (ANS) and associated systems for regulating emotional responses such as self-compassion.^38^ The facilitator explores the concept of psychological flexibility, the ability to recognize and adapt to situational demands in pursuit of longer-term outcomes, which has been associated with lower COVID-19 related stress during lockdown^39^ and improved outcomes in ME/CFS.^40^ The session ends by considering stress management strategies that may help participants better cope with their persistent symptoms, build resilience, and promote self-compassion. The workbook builds upon these stress management principles, encouraging participants to consider the key points in relation to their current stressors. The workbook draws upon third wave cognitive behavioral therapies (CBT) such as acceptance and commitment therapy (ACT),^41^ compassion focused therapy (CFT),^38^ mindfulness-based cognitive therapy,^42^ and polyvagal theory^23^ to consider bi-directional pathways between the mind and body, demonstrating how stressors affect participants’ thoughts, feelings, behaviors, physical sensations, and energy levels. These impacts are linked back to the automatic nervous system (ANS), and participants are supported to develop effective stress management strategies that activate the more “soothing system” parasympathetic (ventral vagal) nervous system.^38^

### Week 7: What Next? Planning for the Future

The final session is facilitated by an occupational therapist, clinical psychologist, personal support navigator, and assistant psychologist. It establishes relapse as part of the recovery process, with participants encouraged to reflect upon the setbacks they have already experienced. This session focuses on the lessons that can be learned from symptom flare ups and strategies for managing them, revisiting the principles that underpin activity management and energy conservation. The session ends with an overview of questions that can help guide participants to create an action plan for overcoming setbacks and integrating lessons learned following a relapse. The workbook considers these strategies in more detail and includes a template setback recovery plan for participants to complete. Participants are also provided with resources signposting them to further support if symptoms persist, with details of additional services NHS and voluntary services locally and nationally. Throughout the 7-week course, participants are encouraged to see their GP following any new symptoms to rule out malignant causes. Participants are also encouraged to request a full blood count via their GP, as well as vitamin D, B12, and Ferritin testing, if this has not yet been performed in order to highlight other causes/contributing factors for symptoms.

## “*Recovering From COVID*” Service Evaluation Methods

The EuroQol EQ-5D-5L assesses health-related quality of life (HRQoL) across five dimensions, including mobility, self-care, usual activities, pain/discomfort, and anxiety/depression.^43^ Each dimension is scored from 1 (no problems) to 5 (extreme problems). These scores yield over 3000 possible health states, ranging from “full health” (ie, 11 111) to “worst health” (ie, 55 555). The scores from all 5 dimensions are combined and scaled, based on UK norms, to provide an index that represents overall HRQoL ranging from −0.594 to 1 with 1 the best possible quality of life. In addition, there is an overall health self-report visual analog scale (VAS) rating from 0 to 100 where 100 represents the best possible health. Individuals enrolled on the “*Recovering from COVID*” course were invited to complete the EQ-5D-5L to report their health prior to COVID-19 (*Pre-COVID*) as well as their current state of health at the time of course enrollment (*Pre-course*) using Microsoft Forms. This was completed approximately 1 to 3 weeks before the first session. Subsequently, participants were invited to complete the EQ-5D- 5L between session 6 and 7 during the rehabilitation course (*Post-course*). Participants who did not complete the EQ-5D-5L at week 7 were sent 2 reminders; 1 and 2 weeks after the last course session. From the 149 participants who completed the pre-course assessments, 76 participants responded to the post-course assessments. We ran Little’s Missing Completely at Random (MCAR) test^44^ to establish that data were missing at random which showed a trend toward significance (*P* = .059). Missing data was not estimated and analysis was ran using the 76 participants who completed all three assessments. In this subsample we encountered 5% missing data for the VAS and Index scores. We obtained estimates in the presence of missing data using the expectation maximization (EM) algorithm.^45^ Repeated measures analysis of variance (ANOVAs) were computed using SPSS (Version 27) with 3 levels of assessment (Pre-COVID, Pre-Course and Post-Course) with statistical significance set to *p* < .05 using Bonferroni correction. Multiple regression analyses were also conducted for VAS and Index scores to investigate whether age and symptom duration significantly predicted scores health improvements.

## Participants

Two hundred and nineteen participants registered for the “*Recovering from COVID*” course. Given that testing for acute COVID-19 was not widely available at the beginning of the pandemic, an inclusive approach was taken by allowing self-diagnosis of suspected acute COVID-19 and “Long-COVID” based on symptoms in line with NICE rapid guidance. Specifically, recruitment targeted “social, health and care staff with suspected long-COVID.” Recruitment was opportunistic by reaching out to key stakeholders within the 3 local NHS Foundation Trusts covering Bradford, Airedale, and Craven (BDCFT, Bradford Teaching Hospitals NHS Foundation Trust, Airedale NHS Foundation Trust), including HR and infection control, the Clinical Commissioning Group, managers and contacts within the local councils, voluntary sector, and GP practices through the primary care network. All participants were required to have access to digital technology (ie, laptops, smartphones, tablets) to register for the course and were provided support for accessing and using Microsoft Teams.

One hundreds and forty nine participants provided demographic information and completed baseline and pre-course assessments including 112 females and 16 males (21 missing gender) with a mean age of 47.25 (10.44) years. 67% identified as White British, 13% as South Asian, 4% Asian British, 3% Mixed heritage, and 13% missing. In 2020, BDCFT published a report stating that 81% of their workforce is female and 21% of their staff are from black, Asian or ethnic minority (BAME) backgrounds,^46^ suggesting that these characteristics are representative of staff in this district. Subsequently, 76 participants completed the post-course assessment including 53 females and 8 males (15 missing gender) with a mean age of 48.97 (10.25) years. 79% identified as identified as White British, 7% as South Asian, 3% Asian British, 4% Mixed heritage, and 7% missing. All participants lived and/or worked in the Bradford, Airedale, and Craven district whereby 80% of participants were social, health, and care staff working across Bradford, Airedale, and Craven.

## Results

Overall, 12.8% of participants had been admitted to hospital during the acute phase of COVID-19 for a mean of 12.32 days (S.D. 18.58). The mean time elapsed between onset of symptoms and enrollment in the “*Recovering from COVID*” course was 5.99 months (S.D: 3.89; range 1-16). 60.5% reported a positive COVID-19 test at the time of acute infection, although testing was not widely available when many of the sample first experienced symptoms. Only 25% of participants were working as usual whereas 38% were on long-term sickness leave and 17% on a phased return.

Attendance for the course was very good and 92.1% of participants attended at least 4 times out of the 7-week course with 74.5% attending at least 6 times. Evaluation of participant feedback (n = 76) showed that 96% of people felt more knowledgeable and informed about their symptoms, 100% felt the exercises throughout the course were helpful, 91% felt confident in implementing strategies to manage symptoms, and 99% of participants would recommend the course to others.

Analyses revealed a statistically significant effect of post-COVID syndrome upon VAS scores (*F*(2, 150) = 77.13, *p* < .001, η^2^ = .49) showing a significant decrease from pre-COVID to pre-course assessment of 33% (*F*(1, 75) = 114.20, *p* < .001, ηp^2^ = .60). This was followed by a significant improvement in VAS scores (10%) at the post-course assessment (*F*(1, 75) = 18.22, *p* < .001, η^2^ = .20). Previous studies have considered differences of 7 or more on the VAS to be clinically significant.^47^ Table 1 shows mean scores of VAS and index scores at each time point. We also report overall scores for all participants who completed pre-COVID and pre-course assessments. We observed no statistically significant effects between participants who completed the follow up and those that did not (all *p* > .05).

**Table 1.** Means and Standard Deviations for VAS and Index Scores Reported for Pre-COVID-19, Pre-Course, and Post-Course.

Outcome measures	Time	Full sample (149 participants) Mean (S.D.)	Subset (76 participants) Mean (S.D.)
Overall health (0-100)	Pre-COVID	80.74 (17.76)	81.54 (19.09)
	Pre-course	50.24 (21.91)	48.51 (21.98)
	Post-course	—	58.65 (20.09)
UK Index (0-1)	Pre-COVID	0.87 (0.17)	0.89 (0.17)
	Pre-course	0.55 (0.24)	0.55 (0.22)
	Post-course	—	0.62 (0.22)

**Figure 2.**
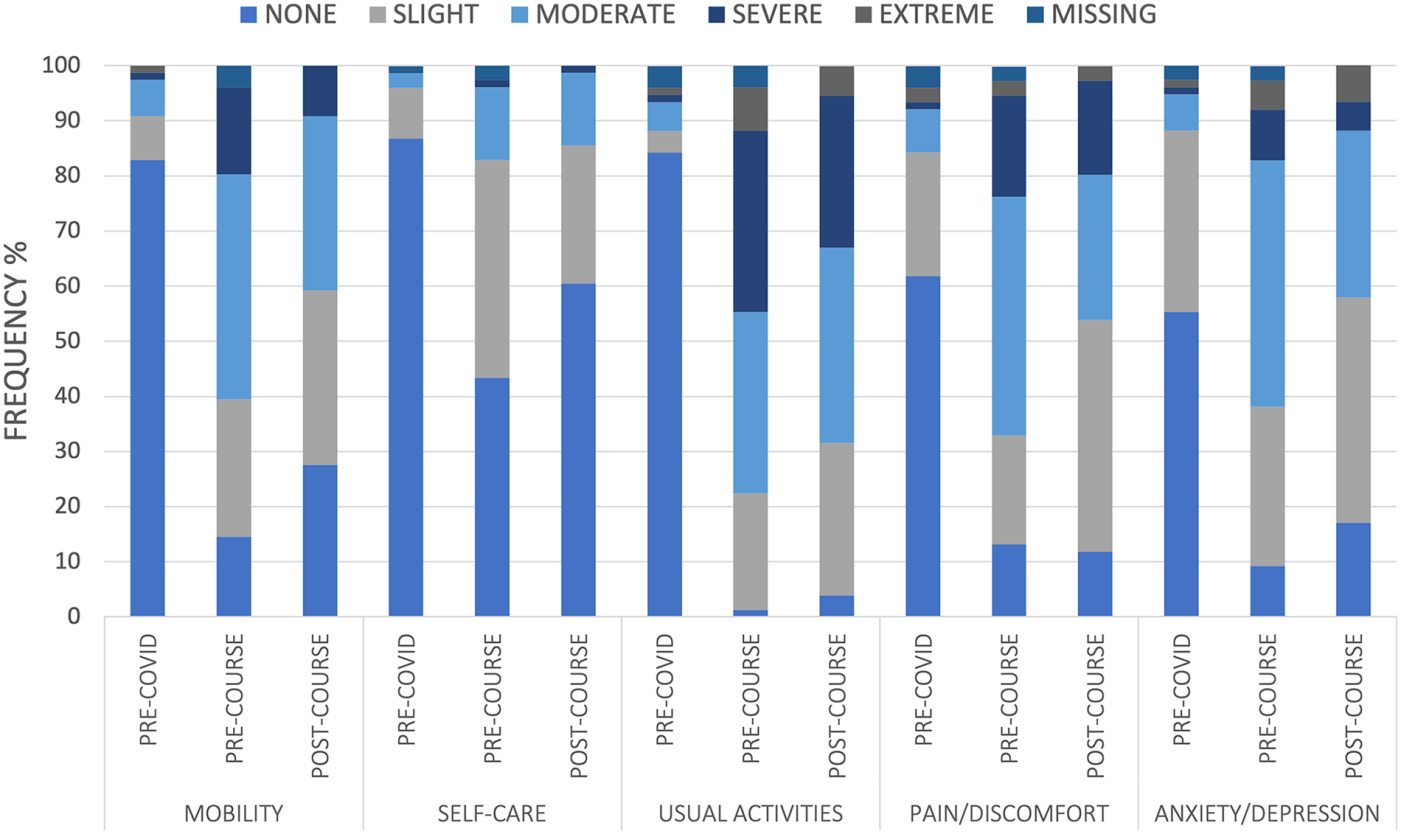
Frequency of problems reported across all 5 domains of the EQ-5D-5L at each time point; Pre-COVID, Pre-Course, and Post-Course.

Similarly, we observed a statistically significant effect of assessment upon UK weighted Index scores (*F*(2, 150) = 97.37, *p* < .001, η^2^ = .57) showing a significant decrease from pre- COVID to pre-course assessment of 34% (*F*(1, 75) = 227.48, *p* < .001, η^2^ = .75). This was followed by a significant improvement in Index scores (7%) at the post-course assessment (*F*(1, 65) = 6.29, *p* < .01, η^2^ = .08). Frequencies of participants reporting problems across the 5 dimensions are illustrated in Figure 2.

We further analyzed the data by categorizing participants into health states. The results indicate that 28 participants (42%) reported the “full health” state prior to COVID-19 with no reported problems across the 5 domains (ie, heath state 11 111). Given that this course was aimed at supporting people reporting post-COVID syndrome, unsurprisingly, this fell to 0% at *Pre-Course* with 3% of individuals reporting being back to full-health *Post-Course*. Following Devlin et al’s^48^ Pareto Classification of Health Change (PCHC) we classified participant’s health states as (1) Their heath state is worse, (2) Their health state is exactly the same, (3) The changes in health are “mixed”: better on one dimension, but worse on another. We observed that at the pre-course assessment 4.5% had no change from their pre-COVID levels, whilst 95.5% of participants exhibited a worse health state. At the post-course assessment 53.9% reported an improved health state, 7.9% had no change, 10.5% reported a worse health state, and 22.4% reported a mixed change in health state. Looking at the dimensions individually, we added a fourth category to account for participants who reported no problems at either time point: (4) Their health was unchanged with no problems pre- and post-course. In Table 2 these participants were recorded as “no change: no problems” whereas participants who did report problems which were unchanged were coded as “no change: problems.”

**Table 2.** Frequency Health State Change Across All 5 Domains of the EQ-5D-5L.

Outcome measures	Health state change	Freq %
Overall heath state	Worse	10.5
	No change	7.9
	Mixed	22.4
	Improvement	53.9
	Missing	5.3
Mobility	Worse	9.2
	No change: problems	30.3
	No change: no problems	10.5
	Improvement	46.1
	Missing	3.9
Self-care	Worse	11.8
	No change: problems	18.4
	No change: no problems	35.5
	Improvement	31.6
	Missing	2.6
Usual activities	Worse	9.2
	No change: problems	48.7
	No change: no problems	0
	Improvement	38.2
	Missing	3.9
Pain/discomfort	Worse	11.8
	No change: problems	39.5
	No change: no problems	6.6
	Improvement	39.5
	Missing	2.6
Anxiety/depression	Worse	13.2
	No change: problems	42.1
	No change: no problems	5.3
	Improvement	36.8
	Missing	2.6

## Participant Characteristics and EQ-5D-5L

To examine the effect of gender, we entered gender (Female = 112; Male = 16) as a between subjects factor in the repeated measures ANOVA, but did not observe any significant difference in either overall VAS or Index scores (both *p* > .05), nevertheless with a substantial gender imbalance, further research is needed to establish potential gender differences which may be of relevance.

Given the critical need to consider the disproportionate mortality and morbidity amongst BAME NHS staff, who have contracted COVID-19,^49^ we entered ethnicity (White = 103; BAME = 36) as a between subjects factor in the repeated measures ANOVA. We did not observe a significant effect of ethnicity on pre-COVID Index scores (*F*(1, 137) = 1.74, *p* = .10, η^2^ = .02), however, we did observe a significant effect of ethnicity on pre-course Index scores (*F*(1, 137) = 5.79, *p* = .017, η^2^ = .04) showing that BAME participants had a 10% significantly lower Index score (Mean = 0.47; SD = 0.28) compared to White participants (Mean = 0.57; SD = 0.22). We observed no significant effect of ethnicity on overall VAS scores. With only 10 BAME participants who completed the post-course assessments, this highlights a desperate need to further investigate outcomes of rehabilitation in BAME individuals.

A multiple linear regression revealed that improvement in overall VAS scores was not significantly predicted by age (β = −.35, *t* = −1.29, *p* = .20) or symptom duration (β = .52, *t* = 0.73, *p* = .47). Similarly, improvements in Index scores were not significantly predicted by age (β = −.003, *t* = −1.08, *p* = .28) or symptom duration (β = −.004, *t* = −0.50, *p* = .62). We also evaluated whether there were any significant differences in outcome measures between those that tested positive for acute COVID-19 infection and those that did not. We found no significant differences in VAS (*F*(1, 76) = 2.85, *p* = .10, η^2^ = .03) or index scores (*F*(1, 137) = 0.08, *p* = .78, η^2^ = .001).

## Discussion

Although several post-COVID-19 syndrome clinics have been established across the UK, and multidisciplinary treatment pathways described,^50^ there remains little evidence concerning the efficacy of rehabilitation services for the long-term effects of COVID-19. Our data shows significant improvements in quality-of-life outcomes following the “*Recovering from COVID*” course offering hope that multidisciplinary rehabilitation is effective in reversing some of the problems faced by people living with the long-term effects of COVID-19.

The “*Recovering from COVID*” rehabilitation course focuses on sleep optimization, nutrition, activity management, energy conservation, stress management, and breathing optimization, and is delivered by an interdisciplinary team of specialists with expertise in rehabilitation and management of CFS/ME and persistent physical symptoms. The course combines self-management, which is undoubtedly critical in recovery from post-COVID-19 syndrome, with specialist support to educate, motivate, and support individuals to develop personally tailored strategies to manage their symptoms. Indeed, building individuals’ knowledge, skills and confidence to manage their enduring symptoms are well established for long-term conditions and can improve outcomes^51^ but education only programs are the least successful and highlight the importance of support from an interdisciplinary team of healthcare professionals^52^ and peer support.^53^

Although participants showed significant improvements, it is evident that health and quality of life remain significantly affected in post-COVID-19 syndrome with only 3% of participants returning to full health. We advocate that post-COVID-19 syndrome should be considered a long-term health condition that requires continuing support from an interdisciplinary team. Importantly, the research into post-COVID-19 syndrome and the ongoing research into ME/CFS may have a symbiotic relationship, with advances made in each medical illness able to benefit patients suffering from post-COVID-19 syndrome and ME/CFS.^18^ Whilst we hope that this will lead to increased funding for research into post COVID-19 syndrome and CFS/ME, and ultimately a cure, evidence-based lifestyle management programs delivered by experienced and skilled health care professionals can offer some hope by optimizing the health of people living with these conditions. An economic evaluation was not completed for this service; however, it is important to note the potential cost and workforce implications for NHS front line staff with post-COVID syndrome in the context of the current “NHS workforce Crisis” Kings Fund 2021 and the importance of rapid access to an effective intervention, such as the “*Recovering from COVID*” course.

The PCWBS, located within primary care, have been promoting holistic, interdisciplinary, and multi-agency working since its conception in 2016. The PCWBS is an award-winning psychology led service and has been highlighted by The King’s Fund as a vanguard site for new models of care in mental health^54^ and an innovative site providing creative solutions for longstanding problems and gaps in support by the Centre for Mental Health.^55^ The Kings Fund highlights that mental health care is often disconnected from the wider health and social care system—institutionally, professionally, clinically and culturally, whereby many people do not receive co-ordinated support for their physical health, mental health, and wider social needs.^54^ Clinical psychologists may be particularly well placed to provide leadership in new models of care due to their extensive training in the understanding of human behavior, communication, engagement, collaboration, and research.^56^

## Limitations

The creation of the “*Recovering from COVID*” course was a rapid response to increasing concern about the long-term effects of acute COVID-19 infection, and consequently, there are a number of limitations. Firstly, data from a comparison group was not obtained to evaluate the extent to which symptom improvement can be attributed to the rehabilitation pathway. This is an important consideration for future research. Second, response rates for outcome measures were low with only half of participants completing the post-course assessments. It is therefore important to consider potential bias regarding participants who completed the responses and those that did not. This is especially important considering that participants on this course had self-referred to the course and were perhaps more motivated. Similarly, people who did not enroll on the course may have felt too unwell to participate. Further research is needed with ongoing development of suitable outcome measures. Third, the long-term effects of this rehabilitation course are yet to be evaluated and therefore require further data collection and evaluation. Finally, retrospective self-reports of health were used to evaluate pre-COVID health. Despite numerous studies supporting the use of retrospective measurement of baseline data following a health event or an intervention,^57^ caution should be taken in interpreting retrospective pre-COVID reports of health.^58^

## Future Directions

Given that we observed a significantly greater self-reported health decline in BAME staff but had insufficient data to evaluate differences in improvement at the post-course assessment, there is a critical need to further investigate post-COVID syndrome in BAME staff and the wider population. In addition, it is also important to evaluate what proportion of people with post-COVID syndrome would meet the criteria for CFS/ME.

Overall, the PCWBS’s approach remains consistent with the latest research in post- COVID syndrome, but nevertheless, learning and development is continually required. For instance, when the “*Recovering from COVID*” course was first piloted, the PCWBS did not have access to a speech and language therapist and therefore, in the following course, participants were subsequently screened for speech and language therapy input and handouts were sent retrospectively. Similarly, as more information was gathered from research, some slides used in the course were updated. Further development and roll-out of this course will be guided by evidence-based research as it becomes available. The PCWBS is currently developing a number of brief course interventions including a 5-week “*Back to Baseline course*” lead by an occupational therapist and a physiotherapist to look more closely at activity management as well as a *3-week breathing course*, given that a high proportion of participants were presenting with dysfunctional breathing patterns. The PCWBS is also piloting a *6-week self-compassion course* due to evidence of perfectionism/high achievers being more vulnerable to developing CFS/ME.^59^ Consistent with this, individuals participating in the “*Recovering from COVID*” course showed signs of high expectations and high levels of self-criticism. Anecdotally, this appeared to have a direct link to individuals finding it hard to pace activity management despite having the practical knowledge and information. Future studies may reveal this to be a significant marker of vulnerability and therefore an area to be targeted for effective intervention.

## Summary

In summary, the “*Recovering from COVID*” course offers hope that multidisciplinary rehabilitation is effective in reversing some of the long-term effects of an acute COVID-19 infection. We anticipate that the COVID-19 pandemic will further facilitate holistic and integrated physical and mental health care systems and provide a blueprint for rehabilitation services across the country, not only regarding the treatment of post COVID-19 syndrome, but to develop and transform health care across all chronic, enduring, and long-term health conditions.
